# Editorial: Analysing emotional labor in the service industries: consumer and business perspectives, volume II

**DOI:** 10.3389/fpsyg.2025.1600934

**Published:** 2025-04-09

**Authors:** Weon Sang Yoo, JungKun Park, Ki-Joon Back

**Affiliations:** ^1^School of Business, Korea University, Seoul, Republic of Korea; ^2^School of Business, Hanyang University, Seoul, Republic of Korea; ^3^College of Global Hospitality Leadership, University of Houston, Houston, TX, United States

**Keywords:** emotional labor, service industries, leadership, team norms, workplace relationships

Emotional labor has emerged as a defining element of service work in both B2C and B2B contexts (Zhao et al., 2020; Humphrey, 2023; Lin et al., 2025). It involves the management of emotions by employees to meet organizational expectations and deliver satisfactory service experiences to customers. As organizations increasingly rely on service excellence for competitive differentiation, emotional labor is not just an individual-level psychological phenomenon—it becomes a strategic asset and a source of competitive advantage (Klein, 2021; Park et al., 2021). Volume II of the Research Topic “*Analysing Emotional Labor in the Service Industries: Consumer and Business Perspectives*” builds upon the insights from the first volume and brings together six empirically grounded studies that deepen our understanding of emotional labor through multiple lenses: leadership, team norms, healthcare settings, digital reviews, workplace relationships, and emotional authenticity.

Cheng et al. explore the interplay between empowering leadership and frontline employees' emotional labor, focusing on the mediating role of job passion. Drawing on data from over 1,000 service employees in China, the authors found that empowering leadership—which promotes autonomy and a sense of control—significantly increases deep acting and decreases surface acting. Interestingly, harmonious passion mediated positive effects on deep acting, while obsessive passion presented a more complex “double-edged” role. The findings highlight how leadership can shape emotional authenticity in service work, suggesting that effective managers should nurture environments that foster intrinsic motivation rather than compliance-driven behaviors.

Gündüz Çekmecelioǧlu et al. examine the healthcare sector, a context where emotional labor is particularly intense due to the high demands placed on service workers. Using the Job Demands-Resources (JD-R) and Conservation of Resources (COR) theories as a framework, the study investigates how organizational impediments—such as insufficient resources, bureaucratic constraints, and poor communication—affect healthcare workers' emotional regulation and job satisfaction. The results indicate that both surface and deep acting increase under such stressors, but only surface acting significantly mediates the negative impact on job satisfaction. The implication is clear: surface acting may be a short-term coping mechanism but is detrimental in the long run. These findings provide actionable insights for healthcare leaders aiming to support emotional wellbeing through structural reforms and resource provision.

Hong and Kim delve into emotional labor within teams during a shift from customer-focused emotional labor. This study looks at how shared emotional display norms within teams influence individual emotion regulation strategies. Using multilevel modeling, they show that employees are more likely to engage in surface acting when there is a mismatch—emotional dissonance—between an individual's internal state and the team's emotional norms. Moreover, regulatory focus (promotion vs. prevention) moderates this relationship, further complicating the emotional calculus within team dynamics. The study also finds that surface acting negatively affects selfless organizational citizenship behavior (OCB), signaling broader organizational costs. These results are particularly relevant for team leaders and HR practitioners seeking to foster inclusive emotional climates.

Li-na et al. provide a digital-era perspective by investigating how consumers express discrete emotions in online product reviews. Using sentiment analysis and human behavior dynamics, the study analyzes time patterns in consumer emotions following purchases. Emotions such as satisfaction, admiration, reproach, and hate show distinct temporal behaviors, often peaking immediately after purchase and again after ~11 days. The bimodal nature of these emotional expressions suggests that consumer feedback is shaped not only by initial impressions but also by delayed evaluations, possibly influenced by ongoing product usage or delayed delivery experiences. These insights are highly relevant to marketers, UX designers, and e-commerce platforms, as they underscore the importance of timing in interpreting consumer sentiment and implementing service recovery strategies.

In another emotionally charged service environment, Li et al. investigate the emotional labor strategies of operating room nurses and their impact on work-related quality of life (WRQoL). The study conducted across 11 hospitals in China reveals that expressing naturally felt emotions contributes most positively to WRQoL, followed by deep acting. Surface acting, conversely, has a significantly negative association. The findings offer vital guidance for hospital administrators and nursing managers: encouraging authenticity and providing emotional support resources can significantly enhance nurse satisfaction and reduce burnout. Given the global shortage of nurses and the rising stress levels in healthcare, these findings hold practical and policy relevance.

The final contribution by Kim and Oh brings a social network perspective into the emotional labor discussion. Integrating social network theory with COR theory, the authors argue that employees with central positions in workplace friendship networks are better equipped to perform deep acting. These employees benefit from psychological resources such as positive affect and elevated self-perception. Across a multi-study design, they show that friendship network centrality is positively associated with the intention and ability to engage in authentic emotional displays. This study reframes emotional labor not only as an individual or managerial issue but as a structural phenomenon embedded in the social fabric of the workplace.

The six studies in this volume reflect the growing sophistication of emotional labor research. Several cross-cutting themes emerge. First, the critical role of leadership and management in shaping emotional labor strategies cannot be overstated. Whether it is empowering leadership in service firms or ineffective structures in healthcare, managerial practices directly affect how employees manage their emotions. Second, the emotional dynamics of peer interactions are just as influential as those of customer-facing roles. Emotional display norms, friendship networks, and team climates all interact to either facilitate or hinder authentic emotional expression. Third, digital and asynchronous service interactions—like online reviews—present new avenues for studying emotional labor from the consumer perspective, emphasizing the need for interdisciplinary research that blends psychology, marketing, and data science.

For practitioners, these findings offer a roadmap for building emotionally intelligent organizations. Emotional labor is not simply a personal burden for employees to bear. It is a relational and organizational issue that requires strategic attention. Interventions could include leadership development, redesigning work environments to reduce emotional dissonance, enabling team-based emotional intelligence training, and fostering social support networks at work. Platforms handling consumer feedback can also benefit from sentiment analysis and behavioral analytics to better understand the emotional trajectories of their customers.

In conclusion, this second volume of the Research Topic significantly advances our understanding of emotional labor in modern service industries. It showcases how emotional labor is both shaped by and shapes broader organizational structures, interpersonal dynamics, and consumer behavior. We hope this Research Topic encourages further research, policy innovation, and managerial action to promote emotional resilience, authenticity, and wellbeing in service work.
